# Patient Attitudes Toward Mobile Device Use by Health Care Providers in the Emergency Department: Cross-Sectional Survey

**DOI:** 10.2196/16917

**Published:** 2020-03-31

**Authors:** Mohamad Alameddine, Hani Tamim, Dima Hadid, Mohamad-Ali Cheaito, Maha Makki, Hadi Maatouk, Eveline Hitti

**Affiliations:** 1 Department of Health Management and Policy American University of Beirut Beirut Lebanon; 2 Department of Internal Medicine American University of Beirut Beirut Lebanon; 3 Department of Emergency Medicine American University of Beirut Beirut Lebanon

**Keywords:** smart devices, emergency department, patients, attitudes, digital professionalism, code of conduct, empathy, professionalism, distraction, attention

## Abstract

**Background:**

Health care provider usage of mobile devices is increasing globally; however, there is little understanding of patient perceptions on this behavior in a health care setting.

**Objective:**

The aim of this study was to assess patients’ attitudes toward mobile device usage by health care providers in the emergency department and to identify predictors of these attitudes.

**Methods:**

The study was carried out at the emergency department of a large academic tertiary care medical center in Lebanon. A cross-sectional survey design was adopted by administering a questionnaire to medically stable adult patients who presented to the emergency department with an emergency severity index of 3, 4, or 5 between January 2017 and March 2018. The questionnaire collected relevant patient demographic information and included questions related to their mobile device usage along with those evaluating attitudes for the use of mobile devices by health care providers with respect to six major domains: role in health care, distraction potential, impact on communication, empathy, privacy, and professionalism. The attitude toward mobile device usage by health care providers in the emergency department was the main outcome variable. A stepwise logistic regression model was used to assess the association between the outcome variable and the demographic and attitude-related independent variables.

**Results:**

Among the 438 eligible patients, 338 patients responded to the questionnaire for a response rate of 70.0%. Overall, 313/338 (92.6%) respondents agreed that mobile devices improve health care delivery, whereas 132/338 (39.1%) respondents were opposed to their usage by health care providers in the emergency department (95% CI: 34.0-44.4). The majority (240/338, 71.0%) of patients agreed that mobile devices are a source of distraction to health care providers in the workplace. Females (odds ratio [OR]=1.67, 95% CI: 1.00-2.78) as well as all patients (OR=2.54, 95% CI 1.36-4.76) who believed that mobile devices were a source of distraction, reflecting a lack of professionalism (OR=2.77, 95% CI 1.59-4.82) and impacting the provider’s ability to relate to the patient (OR=2.93, 95% CI 1.72-4.99), were more likely to agree that mobile devices should not be used in the emergency department.

**Conclusions:**

Patients’ negative attitude toward mobile device use in the emergency department is largely driven by patient gender (females), patient perception of the distraction potential of the devices, and their negative impact on the health care provider’s empathy and professionalism. The findings of this study shed light on the importance of encouraging stakeholders to impose a digital professionalism code of conduct for providers working in acute health care settings.

## Introduction

The penetration and usage of mobile devices is increasing globally, with growing penetration into the health care sector. A previous survey indicated that 87% of health care providers report using some form of portable network-enabled electronic device such as smartphones in the workplace, paralleling the rapid growth in health care apps, which is now the third fastest–growing app category on the market [1]. Mobile devices are generally considered to be of value to patients and providers in the form of speed of information transmission, clinical decision making, and accessibility [1,2]; however, little is known about patient perceptions of health care providers’ usage of mobile devices in a health care setting.

Physicians, both senior and those in training, regularly use mobile devices to access medical apps (eg, drug guides, medical calculators), capture work-related images, respond to bleeps, communicate with their teams, and request diagnostics, among other uses [2-5]. In addition, mobile devices increase provider accessibility, improve communication, and help promote collegiality among the health care delivery team [6]. The usage of mobile devices in health care settings is not a passing trend but rather a practice that is now highly integrated into the work culture, which is likely to expand and grow in the future [7].

Despite these advantages, there are concerns about the regular use of mobile devices in health care settings, including the potential of jeopardizing the privacy of patient information, interfering with patient devices (eg, electromagnetic resonance), as well as cross-contaminating patient care areas [8,9]. Furthermore, there is a potential impact of such distraction on clinical care, especially in high-risk areas such as the emergency department characterized by a high cognitive load and regular interruptions. Such distraction potential has serious safety implications, including the risk that physicians and residents will miss vital patient information [8,10]. In fact, approximately 41% of health care providers at the American University of Beirut Medical Center (Beirut, Lebanon) reported distraction by nonwork-related use of mobile devices [11]. There is a growing body of literature on the safety implications of the usage of mobile devices and the so-called “inattentional blindness” associated with their use. However, few studies have directly investigated patient perceptions of their providers’ usage of mobile devices in health care settings or the impact of such use on the physician-patient relationship.

Studies that have examined the effect of mobile device usage on interpersonal relations showed that the presence of mobile devices in a social setting negatively affects the quality of conversations, extent of satisfaction with a social encounter, as well as the level of empathy and connection [12-14]. Several studies have explored patient attitudes toward health technology and their impact on the patient-provider relationship. However, these studies have been limited to the use of computers in the consultation room [15-17], tablets in the examination room and telerounding [18,19], mobile phone images in wound care [20-22], personal digital assistants in emergency departments [23], and personalized smart bedside stations in an inpatient setting [24]. In general, most patients did not express a negative attitude toward their physicians’ use of such technology [18,23,25] and did not feel that their interaction with their care provider was less personal due to the use of the technology [16,18].

However, these findings cannot be extrapolated to the usage of mobile devices for several reasons, including the mobility of such devices, their strong distracting potential, association with the users’ wider social network even when not actively being used, and accumulating evidence on their negative impact on empathy and quality of interactions. Focused assessments of patient attitudes toward their health care providers’ use of mobile devices in health care settings is of importance [12-14], especially in the context of the emergency department. This is because the nature of work in the emergency department poses specific challenges for both safety and relationship building, which is characterized by high volumes, short interactions, and frequent interruptions [26-28]. Therefore, understanding patient perspectives in this setting can guide policy and practice recommendations that will help address patient concerns and preserve the patient-provider relationship as mobile device adoption in health care continues to rise.

Accordingly, the aim of this study was to assess patients’ attitudes toward the usage of mobile devices by health care providers in the emergency department. Moreover, we statistically explored the predictors of these attitudes, including demographic characteristics as well as perceptions of the role in health care, distraction potential, impact on communication, empathy, privacy, and professionalism.

## Methods

### Study Design and Setting

The study was conducted at the emergency department of an academic tertiary-care medical center in Lebanon (American University of Beirut Medical Center, Beirut, Lebanon). The annual census of 2016 showed that the emergency department received 55,000 patient visits, with 70% of patients presenting on weekdays and the remaining presenting on weekends. Trained nurses triage patients based on the emergency severity index (ESI) and age. ESI is a 5-level index used in emergency departments to rate a patient’s acuity from level 1 (most urgent) to level 5 (least urgent) based on an estimation of resources required [29].

Desktop computers are available at the nursing station for electronic-based ordering of labs and diagnostics. Otherwise, all other documentation, including nursing and medication orders, are paper-based. There are no workstations on wheels and no tablets integrated into any of the documentation or ordering workflows. All of the health care providers in this emergency department report bringing a mobile device to work, with 83 out of 97 respondents (86%) reporting use of a mobile device for medical purposes [11].

A cross-sectional survey was administered to adult patients presenting to the emergency department. Data collection was carried out between January 2017 and March 2018. Ethical approval was obtained from the Institutional Review Board of the American University of Beirut that the medical center is affiliated with (protocol number ED.EH.06).

### Recruitment of Participants

The eligibility criteria were patients aged 18 years and older presenting to the emergency department during the study period with an ESI of 3, 4, or 5; present in the emergency department for a minimum of 2 hours; and the primary attending physician determined that they were medically stable. Among the 483 eligible subjects invited to participate in this study, 145 refused to participate, resulting in a total of 338 subjects available for analyses (response rate of 70.0%). The main reasons for refusal to participate included not feeling well enough to participate, lack of interest in the research study, or not having time to participate.

A stratified random sampling design was adopted, in which the strata were defined as weekdays (237/338, 70.1%) vs weekends (101/338, 29.9%). The random aspect of this sampling was achieved by carrying out data collection during different times and days of the week, in which all eligible patients available during the visit were identified using the emergency department dashboard, which is an in-house electronic patient tracking system that includes the patients’ age, gender, ESI, and arrival time.

### Measurements

A mobile device was defined as any handheld portable network-enabled electronic device that is generally connected to other devices or networks via different wireless protocols [30-32], primarily smartphones, which have not been addressed by other studies.

A survey instrument in English was developed to evaluate patients’ awareness and attitudes to the utilization of mobile devices in the emergency department. A review of the published peer-reviewed literature and other surveys examining the use mobile devices was carried out to develop the survey questionnaire used in this study [10]. Upon this review, a preliminary version of the survey was constructed, which was further customized to the institutional setting and reviewed by a group of experts, including a statistician, the director of the Emergency Medicine Department, a health management and policy expert, and a social scientist, to enhance content validity (see Multimedia Appendix 1). The English version of the questionnaire was translated into Arabic and then back-translated to English, and the two drafts were compared for consistency. The preliminary drafts were then pilot-tested among 45 patients (who were excluded from the final analyses) for redundancy, validity, and clarity of the questions and statements. The survey was subsequently revised and modified based on patient feedback. Patients were given the option to choose which version they would like to complete based on their preferences. Before administering the survey, the participants were asked to read and sign an informed consent form.

The survey included relevant patient demographic information (age, gender, level of education, patient arrival time, employment status, and monthly income) and their usage of mobile devices. The questionnaire also included a list of statements graded on a 4-point Likert scale (“disagree,” “strongly disagree,” “strongly agree,” or “agree”) that evaluated patients’ attitude toward the use of mobile devices by health care providers with respect to six major domains: role in health care, distraction potential, impact on communication, empathy, privacy, and professionalism.

The attitude toward the usage of mobile device in the emergency department was the main outcome variable considered in this study. More specifically, the statement was “I believe mobile devices should not be used by health care providers in emergency departments.” Responses were divided into two groups: agree (those who answered “agree” or “strongly agree” to that question) and disagree (those who answered “disagree” or “strongly disagree” to that question).

### Sample Size Calculation

Sample size calculation was carried out considering the primary outcome and based on a previous study carried out in a pediatric teaching hospital in Australia, which reported that 33% of patients were against the use of mobile devices at bedside [33]. A sample of 338 patients was estimated with a 95% CI and 5% margin of error to detect a similar distribution.

### Statistical Analysis

Statistical Package for Social Sciences version 24.0 (SPSS Inc, Chicago, IL, USA) was used for data cleaning, management, and analyses. Descriptive statistics are summarized by the number and percentage for categorical variables. The association between “mobile devices should not be used in the emergency department” and other categorical variables was assessed using the Chi square test. Multivariate regression analysis was performed to adjust for potentially confounding variables. Stepwise logistic regression analysis was used to assess the association between the response to “mobile devices should not be used in the emergency department” as a binary variable (agree versus disagree) with all demographic variables and the statistically significant attitude variables. *P*<.05 was set as the entry threshold of potential predictors into the model, whereas *P*<.10 was set as the threshold for removal from the model. The results are presented as the odds ratio (OR) and 95% CI; *P*<.05 was considered statistically significant.

## Results

Among the 338 respondents, 132 (39.1%) were opposed to the usage of mobile devices by health care providers in the emergency department. Table 1 presents the demographic characteristics of all patients and the self-reported mobile device usage, as well as the association with the main outcome (health care providers should not use mobile devices in the emergency department). Overall, the study sample was relatively young with 174/338 (51.5%) respondents aged 35 years or less, with a slightly higher number of women. The majority of the patients were employed and had completed at least a university degree, with slightly more than half earning more than 2000 USD per month. The large majority of respondents reported owning a mobile device, most commonly a smartphone, with the top uses including messaging apps, phone calls, and social media. Analysis of the association between the main outcome and different variables (demographic and self-reported mobile device usage) revealed gender as the only significant factor, with females more likely to agree that mobile devices should not be used in the emergency department.

**Table 1:** Demographic characteristics, self-reported usage of a mobile device, and their association with main outcome.

Characteristic			Health care providers should not use a mobile device in the emergency department, n (%)				
			All (N=338)	Disagree (n=206)	Agree (n=132)		*P* value
**Demographic**							
	**Gender**						.02
		Male	158 (46.7)	107 (51.9)	51 (38.6)		
		Female	180 (53.3)	99 (48.1)	81 (61.4)		
	**Age (years)**						.53
		<25	81 (24.0)	48 (23.3)	33 (25.0)		
		25-35	93 (27.5)	57 (27.7)	36 (27.3)		
		36-50	73 (21.6)	45 (21.8)	28 (21.2)		
		51-65	52 (15.4)	28 (13.6)	24 (18.2)		
		66+	39 (11.5)	28 (13.6)	11 (8.3)		
	**Education level**						.78
		Less than high school	32 (9.6)	20 (10.0)	12 (9.2)		
		High school graduate	47 (14.2)	26 (12.9)	21 (16.0)		
		University graduate	194 (58.4)	121 (60.2)	73 (55.7)		
		Postgraduate	59 (17.8)	34 (16.9)	25 (19.1)		
	Employed		221 (65.4)	133 (64.9)	88 (66.7)		.74
	**Monthly combined family income (USD)**						.40
		<1000	42 (19.9)	30 (22.7)	12 (15.2)		
		1000-2000	55 (26.1)	34 (25.8)	21 (26.6)		
		2000+	114 (54.0)	68 (51.5)	46 (58.2)		
**Utilization**							
	Own a mobile device		327 (96.7)	200 (97.1)	127 (96.2)		.76
	**Type of mobile device owned**						
		Smartphone	319 (97.6)	195 (97.5)	124 (97.6)		>.99
		Tablet	109 (33.3)	70 (35.0)	39 (30.7)		.42
		Smartwatch/band	27 (8.3)	21 (10.5)	6 (4.7)		.06
		Regular phone	1 (0.3)	1 (0.5)	0 (0.0)		>.99
		Other	95 (29.1)	57 (28.5)	38 (29.9)		.81
	**Reasons for using a mobile device**						
		Phone calls	284 (86.9)	176 (88.0)	108 (85.0)		.30
		Messaging apps	284 (86.9)	175 (87.5)	109 (85.8)		.66
		Social media	205 (62.7)	124 (62.0)	81 (63.8)		.75
		Games	112 (34.3)	76 (38.0)	36 (28.3)		.07
		Browsing the internet	202 (61.8)	120 (60.0)	82 (64.6)		.41

Table 2 presents the descriptive analyses of patients’ attitudes toward the usage of a mobile device by health care providers in the emergency department, as well as the association with the main outcome. The majority of respondents believed that mobile devices play a role in patient care and improve health care delivery, but that they should only be used for medical care purposes. According to the respondents, the top reasons for appropriate use of mobile devices in a health care setting are accessing medical information, sending/receiving medical documents/images, looking up patient information, and communicating via messaging apps.

In addition, two thirds of respondents reported that the use of mobile devices does not demonstrate a lack of professionalism, and more than half believe that the use of mobile devices does not cause a breach of confidential patient information. By contrast, more than two thirds of respondents agreed that mobile devices are a distraction to health care providers in the workplace, half agreed that the use of mobile devices by health care providers leads to poor patient-provider communication, and close to half agreed that the use of mobile devices impacts the ability of health care providers to relate to patients.

Moreover, the large majority of patients who agreed that mobile devices are a distraction to health care providers in the workplace were more likely to agree that mobile devices should not be used. Consistently, most patients who agreed that mobile devices lead to poor patient-provider communication were also more likely to agree that mobile devices should not be used in the emergency department. Moreover, patients who agreed that use of mobile devices impacts providers’ ability to relate to patients, demonstrates lack of professionalism, and causes a breach of confidential information were more likely to agree that mobile devices should not be used in the emergency department. Finally, those who do not like providers using their mobile devices when treating them were more likely to agree that mobile devices should not be used in the emergency department.

**Table 2:** Descriptive analysis of patients’ attitudes toward the usage of a mobile device by health care professionals and association with main outcome.

Attitude				Health care providers should not use mobile devices in the emergency department, n (%)						
				All (N=338)		Disagree (n=206)		Agree (n=132)		*P* value
**Role in health care**										
	Agree that mobile devices play a role in patient care			279 (85.3)		176 (88.0)		103 (81.1)		.09
	**Mobile device functions in hospital setting**									
		Access medical information (general)		249 (76.1)		155 (77.5)		94 (74.0)		.47
		Send/receive medical documents/images		245 (74.9)		155 (77.5)		90 (70.9)		.18
		Look up patient information		213 (65.1)		131 (65.5)		82 (64.6)		.86
		Personal calls		150 (45.9)		95 (47.5)		55 (43.3)		.46
		Messaging apps		153 (46.8)		94 (47.0)		59 (46.5)		.92
		Facebook or other social media		105 (32.1)		58 (29.0)		47 (36.2)		.13
	Mobile devices play a role in improving health care delivery			313 (92.6)		196 (95.1)		117 (88.6)		.03
	Mobile devices should only be used for medical care			296 (87.8)		183 (89.3)		113 (85.6)		.32
**Distraction potential**										
	Mobile devices are a distraction to health care providers			240 (71.0)		126 (61.2)		114 (86.4)		<.001
	Health care providers spend more time on their mobile devices than with me			13 (3.8)		6 (2.9)		7 (5.3)		.27
**Communication and empathy**										
	Mobile device usage by health care providers leads to poor patient-provider communication			170 (50.3)		81 (39.3)		89 (67.4)		<.001
	Health care providers’ mobile devices usage impacts their ability to relate to me			151 (44.8)		61 (29.6)		90 (68.7)		<.001
**Professionalism and privacy**										
	I don’t like health care providers using their mobile devices when treating me			205 (60.7)		93 (45.1)		112 (84.8)		<.001
	Mobile device usage demonstrates a lack of professionalism			109 (32.3)		39 (18.9)		70 (53.4)		<.001
	Mobile device usage causes a breach of confidential information			138 (40.9)		68 (33.0)		70 (53.4)		<.001

Table 3 summarizes the independent factors associated with patients’ believing that mobile devices should not be used in the emergency department. Women were more likely to agree that mobile devices should not be used in the emergency department. In addition, patients who agreed that mobile devices were a source of distraction and those who believed they reflected lack of professionalism were more likely to agree that mobile devices should not be used in the emergency department. Moreover, those who felt that the usage of mobile devices impacted the provider’s ability to relate to them were more likely to agree that mobile devices should not be used in the emergency department.

**Table 3:** Multivariate regression analysis for predictors of the main outcome^a^.

Predictor variable	Health care providers should not use mobile devices in the emergency department (reference: Disagree)		
	Odds ratio ( 95% CI )	*P* value	
Gender	1.67 (1.00-2.78)	.05	
Distraction to health care provider	2.54 (1.36-4.76)	.03	
Demonstrates lack of professionalism	2.77 (1.59-4.82)	<.001	
Impacts health care provider’s ability to relate to me	2.93 (1.72-4.99)	<.001	

^a^The following variables were included in the full model: gender (reference: male); age (reference: <25 years); education (reference: <high school); employed (reference: unemployed); distraction; improves health care delivery; mobile device should be used only for medical care; poor communication; impacts health care providers’ ability to relate to me; lack of professionalism; causes a breach of confidential information.

## Discussion

### Principal Findings

This study represents a rare attempt to examine patient perspectives on the use of mobile devices in an acute health care setting. With the growing body of literature on the distraction potential of mobile devices and their negative impact on interpersonal relationships, understanding patients’ perceptions toward the use of mobile devices by health care providers is important for curbing any unintended consequences of their permeation into health care. The present findings reveal that although the majority of patients agree that mobile devices can improve health care and should be used for medical purposes, many felt that mobile devices should not be used in the emergency department (41%).

The results of this study concur with those of existing literature showing that patients acknowledge the importance of technology usage in health care delivery [15-25]. The surveyed patients had clear views on the use of mobile devices, with the majority stating that mobile devices improve health care delivery (92.5%) and that they should be used for medical care (87.5%). Furthermore, close to three quarters of the respondents believed that physicians use their mobile devices in health care settings to access medical information and send or receive medical documents.

The overall positive attitude expressed by patients is counterbalanced by several concerns. Many patients felt that mobile device usage impacted the providers’ ability to relate to them. This is in contrast to studies that considered the patient perspective on other forms of technology, including computers, tablets, and personal digital assistants, who denied any change or depersonalization in their interaction with physicians using such devices [15-20,24,25]. Most previous studies reported that the use of technology in health care is considered to enhance communication and quality of care [19,21,25]. However, our findings are in line with the psychology literature suggesting that mobile devices may negatively affect relationships by dividing an individual’s attention between an immediate face-to-face interaction and a distant wider social network, even when not being actively used. Studies also show that the mere presence of a mobile device during a paired interaction inhibits the ability to develop closeness and trust, in addition to reducing perceived empathy by the partner [13]. Within the health care context, specific features of mobile devices, compared to other technologies, may accentuate the abovementioned negative feelings for patients. The mobility and accessibility of mobile devices, along with their high distraction and addiction potential [34,35], as well as the reduced visibility of the mobile device screen may all heighten feelings of isolation and suspicions that the mobile device is distracting providers from patient care and the face-to-face interaction [34]. This finding is particularly relevant to the context of emergency departments where rapport building, assessment, and communication about care are all being squeezed into a brief encounter where the provider and patient are often meeting for the first time, in a high-interruption, high-anxiety, and high-risk setting [27,28].

Distraction also emerged as one of the main concerns in our study, with more than two thirds of the respondents reporting that mobile devices may be a source of distraction to health care providers in the workplace (71.1%). This finding is in contrast to studies considering the patient perception of provider usage of computers where distraction did not emerge as a significant patient concern. Our findings instead concur with studies that showed high rates of health care professional self-reporting of distraction by a mobile device [10,15,36]. The link between the use of mobile devices and distractibility has been extensively established, showing an association with reduced reaction time, the worst performance on tasks that require cognitive focus, as well as “inattention blindness”, which is the reduced ability to notice unique and novel stimuli [8,37-41]. This perceived distraction may also add to patient concerns about providers relating to them or displaying empathy during the episode of care.

The abovementioned concerns can explain the fact that 2 out of every 5 patients (41%) felt that mobile devices should not be used in the emergency department. Although age, education, and income level were not associated with this patient opinion, gender did emerge as a significant predictor variable, with women being significantly more likely to disagree with the usage of mobile devices in the emergency department setting. This finding is in line with studies showing that men, as compared to women, are more likely to find talking on their mobile phones in various personal situations more acceptable [42]. Moreover, patient perception of how mobile device usage may influence factors related to the care process was highly associated with the potential impact on the provider-patient relationship. Specifically, those who believed that the use of mobile devices reflected lack of professionalism, distracted from care, and negatively impacted the provider’s ability to relate to them also agreed that mobile devices should not be used in the emergency department. Such findings suggest that providers were visible to patients while using their mobile devices or that such use may have taken place during the clinical encounter with the patient. The use of mobile devices by health care providers during clinical encounters would be perceived by many patients to reflect a lack of professionalism, whether it took place inside the emergency department or any other care setting. However, the physical layout of the emergency department, in which all patient care areas are open to the central health care provider station, may have accentuated this perception since health care providers are continuously visible to patients. The ability to relate to patients, a reflection of empathy, was the strongest driver for disagreeing with usage in the emergency department, corroborating the psychology literature on the impact of using mobile devices on empathy and relationship building [12-14].

This study sheds light on serious patient concerns that warrant consideration as mobile device permeability in health care continues to grow. Multiple sectors have already addressed the distracting potential of mobile devices on safety through regulatory initiatives such as the Distracted Driver Law that prohibits usage while driving and the “Sterile cockpit law” that prohibits pilots and crew members from engaging in any activity during critical phases of takeoff and landing [43]. Although the use of mobile devices has become too intertwined with clinical care for a complete ban to be possible, there is a clear need to place some guidelines surrounding their use in health care and introduce some codes of conduct. From a policy perspective, managers need to ensure the right balance between security and liberty [44]. From a liberty perspective, care providers should have the freedom to use their devices as they deem appropriate. From a security perspective, such use should be regulated to mitigate the negative impact on providers’ productivity, patient safety, and the patient-provider relationship. The tradeoff between security and liberty is inevitable and would call for wider discussions between care providers, administrators, regulatory bodies, and the ministry of health to build support for regulatory policies and procedures that could be endorsed at the national level with the support of concerned stakeholders.

Gill et al [8] proposed several guidelines that aimed at securing institutional networks and regulating the use of mobile devices for nonwork-related purposes. This can be realized through ensuring high-security wifi connections and extending firewalls to identify and control the use of an application on the network. Limiting access to social network websites such as YouTube and Facebook and establishing an intracompany communication network are also possible solutions [8].

Nevertheless, successful interventions cannot solely rely on technological solutions to limit personal use. Developing and nurturing a digital professionalism code of conduct will be essential. Setting expectations for clinical care usage in clinical areas and designating separate hotspots for personal use should be part of this implementation. Raising awareness on the impact of the use of mobile devices on face-to-face interactions, empathy, and communication is also essential, with specific attention to female patients. Similarly, it is important to develop best practices for the use of mobile devices around patients, including maintaining eye contact, and explaining to patients why they are using the device to counter the limited screen visibility and associated suspicions that arise.

Although the external validity of the findings and recommendations of this study are particularly applicable to the emergency department setting, they are also applicable, with some contextualization, to other care areas within a health care institution. Future studies should validate the present findings in other care areas and across other contexts.

### Limitations

The results of our study should be considered in light of its limitations. First, although the research team assured patients with the confidentiality of their responses and that their responses will not impact the care they are receiving, there remains a risk of a social desirability bias with patients potentially modifying their responses to prevent putting the care providers at risk. Second, the cross-sectional nature of the study is only able to discover associations, and it is difficult to establish causal relationships, leading to a risk of possible spurious associations. In addition, we excluded high-acuity patients (ESI 1 and 2), which may have led to response bias. However, patients with an ESI 3, 4, and 5 collectively comprise 80% of our population. Lastly, the nature of the study as a single-center assessment with a specific patient base may affect the generalizability to other patient populations.

### Conclusion

Patients in the emergency department recognize the important role of mobile devices in health care delivery and patient care. Nonetheless, 2 out of every 5 patients believe that mobile devices should not be used in the emergency department. This seems to be driven by gender, with women more likely to disagree with usage in the emergency department, along with patients’ perception of how mobile devices may negatively impact the fundamentals of care and the patient-provider relationship, namely professionalism, provider attention, and their ability to relate to patients. This is particularly important in the emergency department setting, where time constraints challenge a physician’s ability to build a rapport with patients. Accordingly, this study highlights the significance of fostering and cultivating, in consultation with concerned stakeholders, a digital professionalism code of conduct in the emergency department with particular attention to female patients.

## Appendix

Multimedia Appendix 1 English Questionnaire.

## Abbreviations

ESI: emergency severity index
OR: odds ratio
