# Supplementary material for: Patient Attitudes Toward Mobile Device Use by Health Care Providers in the Emergency Department: Cross-Sectional Survey
Source: JMIR Mhealth Uhealth. 2020 Mar 31;8(3):e16917. doi: 10.2196/16917 (PMC7157496; doi:10.2196/16917)
Supplement: Multimedia Appendix 1 [file mhealth_v8i3e16917_app1.pdf]

Institutional Review Board
American University of Beirut
09 AUG 2017
RECEIVED

# THE USE OF SMART DEVICES by EMERGENCY DEPARTMENT CARE PROVIDERS: *A patient's perspective*

As a patient in the Emergency Department at AUBMC we would like to invite you to partake in a survey aimed at understanding your attitudes and awareness towards the performance and impact of smart device use by health care providers in the Emergency Department.

Smart Device: An electronic device generally connected to other devices or networks via different protocols such as Bluetooth-NFC-WiFi-3G-etc. that can operate to some extent interactively and autonomously. 2016. in *collinsdictionary.com*.

Retrieved July 1, 2016 from http://www.collinsdictionary.com/submission/115227/Smart+Device

---

**Survey ID number__________**

**Did the patient refuse to participate?**

a. No
b. Yes, if so why? __________________(non-specific reason)

**Time survey commenced_______ (hr:mins)** **Name of research assistant______________**

## ED Characteristics

a. Patient arrival time______ (hr:mins)
b. Total ED length of stay______ (hr:mins)

## Demographic

1. How old are you?
   [Select which age group this falls into__]
   a. <18
   b. 18-24
   c. 25-35
   d. 36-50
   e. 51-65
   f. 65+
2. Gender:
   a. Male
   b. Female

Institutional Review Board
American University of Beirut
14 AUG 2017
APPROVED

3. What is your highest level of education:
    a. No formal education
    b. Elementary
    c. Middle school
    d. High school
    e. Technical
    f. University
    g. Postgraduate
    h. Other
4. Currently employed
    a. Yes
    b. No
5. What is the total monthly combined family household income (in USD)?
    a. <500
    b. 500-1000
    c. 1000-2000
    d. 2000+
    e. Refused to answer

**Awareness of Smart device Utilization**

6. Do you own a smart device?
    a. Yes
    b. No (skip to question 11)
7. What type of smart device do you use?
    a. A smart phone (iphone, android phone, etc.)
    b. Tablet
    c. Smart Watch/ Smart Band
    d. Regular phone (skip to question 11)
    e. Other
8. What are some of the reasons you use your smart device?
    a. Talk
    b. SMS/WhatsApp or other chat services
    c. Facebook or other social media
    d. Games
    e. Browsing the internet
9. Do you think smart devices have a role in patient care?
    a. No (skip to question 11)
    b. Yes

*Institutional Review Board*
*American University of Beirut*

14 AUG 2017

*APPROVED*

10. For which of the following functions do you think health care providers use their phone in the hospital setting? (Circle all that apply)
   a. Ability to access medical information (general)
   b. Ability to send/receive medical documents/image
   c. Ability to look up patient information
   d. Personal calls
   e. SMS/WhatsApp or other chat services
   f. Facebook or other social media
   g. Other: ___________________

11. Since arriving in the ED have you observed any of the ED providers caring directly for you (nurses and physicians) use their smart devices?
   a. No (skip to question 17)
   b. Yes

12. On average, how many times have you observed any of the ED providers caring directly for you (nurses and physicians) use their smart devices since you entered the ED?
   a. 0
   b. 1
   c. 2-5
   d. >5

13. During your experience at the ED, how did you feel when any of the ED providers (nurses and physicians) used their smart device [circle all that are applicable]?
   a. Annoyed
   b. Anxious
   c. Angry
   d. Disappointed
   e. Happy
   f. Less important
   g. Indifferent
   h. I don't have an opinion (skip to question 17).

14. Have you spoken to the ED providers caring directly for you (nurses and physicians) about your feeling with their use of smart devices?
   a. Yes
   b. No

15. If yes, what was his/her reaction?

Institutional Review Board
American University of Beirut
14 AUG 2017
APPROVED

16. If no, why have you not you spoken to him/her about your feeling?

<u>Attitude to Smart device Usage</u> by health care providers

| | Strongly Disagree | Disagree | Agree | Strongly Agree | Not Applicable |
|---|---|---|---|---|---|
| 17. The use of smart devices by health care providers ~~affects~~ leads to poor patient-provider communication? | | | | | |
| 18. Smart devices are a distraction to health care providers in the workplace | | | | | |
| 19. The use of smart devices play an important role in improving healthcare delivery [includes fast lab results, x-ray results] | | | | | |
| 20. I don't like the idea of a health care provider using their smart devices when treating me | | | | | |
| 21. I see health care providers spend more time on their smart devices than with me. | | | | | |
| 22. Smart devices can cause a breach of confidential patient information. | | | | | |
| 23. It is appropriate to you if health care providers used smart devices only for medical care | | | | | |
| 24. Health care providers use of smart devices demonstrates lack of professionalism | | | | | |
| 25. The use of smart devices impacts health care providers' ability to relate to me as a patient | | | | | |
| 26. I believe smart devices should not be used by health care providers in emergency departments | | | | | |

*Institutional Review Board*
*American University of Beirut*

14 AUG 2017

*APPROVED*

27. Do you have any other comments related to clinical staff's use of smart devices?

_______________________________________________________________

_______________________________________________________________

Institutional Review Board
American University of Beirut

14 AUG 2017

APPROVED
